# Container-aided integrative QTL and RNA-seq analysis of Collaborative Cross mice supports distinct sex-oriented molecular modes of response in obesity

**DOI:** 10.1186/s12864-020-07173-x

**Published:** 2020-11-03

**Authors:** Ilona Binenbaum, Hanifa Abu-Toamih Atamni, Georgios Fotakis, Georgia Kontogianni, Theodoros Koutsandreas, Eleftherios Pilalis, Richard Mott, Heinz Himmelbauer, Fuad A. Iraqi, Aristotelis A. Chatziioannou

**Affiliations:** 1 Division of Pediatric Hematology-Oncology, First Department of Pediatrics, National and Kapodistrian University of Athens, Athens, Greece; 2 Department of Biology, University of Patras, Patras, Greece; 3 Department of Clinical Microbiology and Immunology, Sackler Faculty of Medicine, Tel-Aviv University, Tel-Aviv, Israel; 4 Division of Bioinformatics, Medical University of Innsbruck, Innsbruck, Austria; 5 e-NIOS PC, Kallithea, Athens, Greece; 6 Center of Systems Biology, Biomedical Research Foundation of the Academy of Athens, Athens, Greece; 7 Department of Genetics, University College of London, London, UK; 8 Institute of Computational Biology, Department of Biotechnology, University of Life Sciences and Natural Resources, Vienna (BOKU), Vienna, Austria; 9 Centre for Genomic Regulation (CRG), Barcelona, Spain

**Keywords:** Collaborative Cross, Obesity, Sex-differences, High-fat diet, QTL, RNAseq

## Abstract

**Background:**

The Collaborative Cross (CC) mouse population is a valuable resource to study the genetic basis of complex traits, such as obesity. Although the development of obesity is influenced by environmental factors, underlying genetic mechanisms play a crucial role in the response to these factors. The interplay between the genetic background and the gene expression pattern can provide further insight into this response, but we lack robust and easily reproducible workflows to integrate genomic and transcriptomic information in the CC mouse population.

**Results:**

We established an automated and reproducible integrative workflow to analyse complex traits in the CC mouse genetic reference panel at the genomic and transcriptomic levels. We implemented the analytical workflow to assess the underlying genetic mechanisms of host susceptibility to diet induced obesity and integrated these results with diet induced changes in the hepatic gene expression of susceptible and resistant mice. Hepatic gene expression differs significantly between obese and non-obese mice, with a significant sex effect, where male and female mice exhibit different responses and coping mechanisms.

**Conclusion:**

Integration of the data showed that different genes but similar pathways are involved in the genetic susceptibility and disturbed in diet induced obesity. Genetic mechanisms underlying susceptibility to high-fat diet induced obesity are different in female and male mice. The clear distinction we observed in the systemic response to the high-fat diet challenge and to obesity between male and female mice points to the need for further research into distinct sex-related mechanisms in metabolic disease.

**Supplementary Information:**

The online version contains supplementary material available at 10.1186/s12864-020-07173-x.

## Background

The Collaborative Cross (CC) is a multiparent genetic reference panel (GRP) of recombinant inbred lines (RIL) of mice derived from eight different founder strains. The CC resource was developed to facilitate the study of the genetic basis of complex traits, and serve as a uniquely powerful resource for the mapping and integration of various phenotypic and genotypic data [1]. CC lines have been shown to be highly variable for traits related to both normal physiology and disease and have been used successfully in multiple system genetics studies. The CC panel has been the catalyst for the development of a variety of bioinformatics tools for haplotype inference and reconstruction, and genetic mapping [2–4].

Based on a recent report, people with overweight or obese phenotype account for almost two thirds of the population in the USA [5]. The latest estimates in European Union countries show that 30–70% of the adult population are overweight and 10–30% are obese. Some medical associations classify obesity (defined as BMI ≥ 30) as a disease. The development of obesity is affected by various environmental factors such as excess high fat/carbohydrate enriched food consumption and sedentary lifestyle. However, underlying genetic mechanisms are involved in determining the host response to these factors with the rate of heritability of body mass index (BMI) ranging from 40 to 70% in various studies. The CC panel is an experimental population that aids with the dissection of the genetic mechanisms underlying susceptibility to complex traits, such as obesity. Chromosomal regions that are involved in the susceptibility or resistance to the trait can be mapped as quantitative trait loci (QTL) with high precision. QTL mapping results can be strengthened and enriched through integration of RNA-seq data to identify gene expression differences in susceptible versus resistant individuals.

High-throughput sequencing technologies produce millions of reads in a relatively short time, overcoming the limitations of previous technologies and unravelling previously inaccessible complexities in the transcriptome. However, the overall high complexity of the produced datasets, due to their large size and low signal-to-noise ratio, hinders the interpretation of the underlying information. Most recent analytical methods depend on individual tools that users must download, install and use on their physical drives. The process of deciding which bioinformatic tool accommodates the needs of researchers (depending on the experimental approach, the scientific question of interest, as well as the computational needs) can be time-consuming and requires expertise.

Our main aim, was to develop an easy-to-use, scalable and cost-effective workflow for the integrative analysis of genotyping and RNA-seq data from CC mice, offering cross-platform portability to different high-end computing configurations. We then opted to use this workflow to assess the underlying QTL that influence genomic susceptibility to high-fat diet induced obesity in mice and the hepatic gene expression response of the mice to high-fat diet and obesity.

The tools used in each step of the developed workflow are connected through Python scripts, are archived in our public GitHub repository and can be fully modified. The Python scripts are constructed in a user-friendly manner, so that inexperienced users are required to input only the basic parameters (such as the paths to input files, indexes and annotation files) and the whole process is executed automatically, assuming default parameters. More advanced users can apply different sets of input commands or fully modify the scripts according to their requirements.

All bioinformatic tools comprising this workflow have been containerised using Docker containers, resulting in a portable infrastructure that can be executed on physical drives or cloud servers. The main advantage of containerisation is that it is no longer required to install numerous pieces of software, with complex dependencies, downloading instead a pre-built and ready-to-run image file, containing all necessary software and their required dependencies. Another advantage is that applications are run in an isolated and sanitised container environment, where all dependencies are configured for optimal performance, preventing conflicts with other installed programs in the hosting environment. Containerisation ensures the standard operation and performance of applications, not affected by system updates or programming errors from the host end, making the process transparent to the end-user and consistenly fully reproducible. Furthermore, each Docker container can be used as a stand-alone version of the tool making the workflow scalable and adaptable to the individual needs of the researcher.

A question that arises is to what extend the start-up, deployment and instantiation of containers performed by the Docker daemon affect the overall performance and the computational cost of a workflow. An IBM research study suggests that the overhead for CPU and memory performance introduced by Docker containers is negligible, and that containerised applications perform equally or better when compared to virtual machine technology [6]. Regarding the containerisation of genomic pipelines, it has been suggested that Docker containerisation has a negligible impact on the execution performance of common genomic pipelines, especially when tasks (such as the alignment of reads to a reference genome) are generally very time consuming [7].

## Methods

### Experiments

#### Breeding and housing

The Collaborative Cross mouse lines are novel and highly genetically diverse mouse resource population derived from a genetically diverse set of eight founder mouse strains (A/J, C57BL/6 J, 129S1/SvImJ, NOD/LtJ, NZO/HlLtJ, CAST/EiJ, PWK/PhJ, and WSB/EiJ), designed specifically for complex trait analysis, with the aim to overcome the limitations of previously available resources [8]. A cohort of CC lines was developed and currently is housed at conventional environmental conditions at the small animal facility of Tel-Aviv University (TAU) between generations of G27 to G64 of inbreeding by full-sib mating [9]. The CC lines have been maintained and bred by our team at TAU since 2006 as described in the 2008 paper by Iraqi and colleagues [10]. The study was approved by the Institutional Animal Care and Use Committee (IACUC), with the approval numbers M-12-025 and M-14-007.

Mice were weaned at 3 weeks old, housed separately by sex, with a maximum number of five mice per cage, fed with standard rodents’ chow diet (TD.2018SC, Teklad Global, Harlan Inc., Madison, WI, USA, containing % Kcal from Fat 18%, Protein 24%, and Carbohydrates 58%) and water ad libitum. All animals were housed at the TAU animal facility at conventional open environment conditions, in clean polycarbonate cages with stainless metal covers, and bedded with wood shavings, at light:dark cycles of 12:12 h, and constant room temperature of 22^0^_c_ (±2). Due to genetic variations between CC lines, breeding rate, the number and sex of litters in each cycle might vary. Therefore, the CC lines were assessed based on litter availability, while making our best efforts to scan as many of the CC lines as possible with representation of both sexes.

##### Study cohort

The study cohort consisted of 540 mice, from 60 different CC lines that were generated, maintained and studied at the TAU small animal facility. Forty-three of lines had representation of both sexes. A subgroup of 84 mice from 43 CC lines, with 20 lines with representation of both sexes, was selected for RNA sequencing.

#### Dietary challenge

At the age of 8 weeks old, the baseline body weight was recorded. The average body weight (±SE) of females was 18.92 g (±0.27) and of males 22.92 g (±0.24) [11]. Thereafter, the mice were switched to a high-fat diet (HFD) (TD 88137 Harlan Teklad, Madison, WI, USA; containing 42% of calories from fat and 34.1% from carbohydrate, primarily sucrose) starting the dietary challenge for a period of 12 weeks with free access to food and water. During the experiment, mice welfare and health status were monitored daily. The experiment was terminated (euthanasia) for mice that showed deteriorating health, manifested in phenomena such as limited movement, heavy weight loss (about 10% between the weekly weigh measures and over 20% of the initial body weight), apathy and lack of physical activity, interrupted equilibrium (physical instability), breathing difficulty, exceptional behaviour (high aggressiveness / loneliness), and extremely high glucose levels (> 400 mg/dL).

##### Phenotyping

Mice were weighed at the beginning of the HFD challenge and bi-weekly thereafter for the following 12 weeks. Mice that gained less than 10 g to their initial body weight over the dietary challenge period were considered “Normal”, while those that gained over 10 g of body weight were considered “Obese”. The median weight at the beginning of the dietary challenge and the median body weight gain for each CC-line are presented in Supplementary Table 1. At the end of the 12-week dietary challenge, an intraperitoneal glucose tolerance test (IPGTT) was performed after 6 h of fasting to evaluate the diabetic stage of the mice. By using the method of glucose IP injection, the gut effect was bypassed and the glucose stimulated insulin secretion was lower compared to oral administration of glucose. The IPGTT lasted 180 min, with glucose levels measured at different time points before and after the glucose administration. Glucose tolerance was calculated as the total area under the curve between the initial and end time point of the test, for each CC line, separately for females and males. Mice that reached the end time-point of IPGTT with glucose levels under 400 mg/dL were considered “Nondiabetic”, while mice with glucose levels over 400 mg/dL were labelled as “Diabetic”. The complete CC line, sex, baseline body weight at the start of the dietary challenge, body weight gain after 12 weeks of dietary challenge, and blood glucose levels at 180 min after the IPGTT challenge data included in the analysis are presented in Supplementary Table 2.

##### RNA extraction

After overnight recovery from the IPGTT, mice were sacrificed by cervical dislocation, and their livers were collected and stored in liquid nitrogen (− 80 °C). RNA extraction was performed using the QIAGEN commercial kit (Cat.No.73404). Quality control of RNA samples was performed with 2100 BioAnalyzer (Agilent). The RNA Integrity Number (RIN) was used to estimate the integrity of the total RNA sample, samples with RIN above 7.0 passed the quality control test.

##### RNA-seq libraries

RNA-seq libraries were prepared using the TruSeq Stranded mRNA library preparation kit (Illumina). Libraries were pooled and sequenced on the Illumina HiSeq 2000 and 2500 sequencers with Illumina v3 sequencing chemistry. Paired-end sequencing was performed by reading 50 bases at each end of a fragment. Overall, each sample consisted of 24 M to 37.5 M RNA-sequencing fragments with an average of 31.5 M fragments.

##### *RNA-seq* analysis

The base images for the Docker containers were pulled from the repositories of Biocontainers and Rocker [12]. For the purposes of differential expression testing and QTL analysis we developed Docker containers with integrated R v.3.4.2 and all the required packages installed (such as Bioconductor, EdgeR and HAPPY). The Docker images also contained ready-to-run R scripts set on the default parameters so that users unfamiliar with R programming can perform the analyses using R packages otherwise unavailable as stand-alone versions (EdgeR and HAPPY), while advanced users can modify the scripts in a sanitised Docker container environment. In order to run the workflow we developed, the user is required to install the docker engine locally and then pull the images from the Docker Hub repository. This task is relatively fast and requests minimal programming knowledge in comparison to the skills needed for downloading and installing a combination of several pieces of third-party software, while configuring their implicit dependencies and libraries.

##### Quality control

For quality assessment of the reads we used the FastQC tool [13], which is the golden standard for quality control workflows. We did not incorporate a trimming tool in our workflow as aggressive trimming of reads has been suggested to alter RNA-seq expression estimates and the soft-clipping performed by HISAT2 makes read trimming not strictly necessary [14]. However, we provide a docker container with Trim Galore!, which is a tool that makes use of the publicly available adapter trimming tool Cutadapt [15] and FastQC for optional quality control [16].

##### Mapping of reads

Mapping of the reads was performed using the HISAT2 tool, which is a fast and sensitive alignment program for mapping RNA-seq reads [17]. The high efficiency of HISAT2 is based on the indexing scheme it utilises (employing two types of indexes based on Burrows - Wheeler transform and the Ferragina-Manzini index), allowing the tool to perform alignments very fast and with equal or better accuracy than any other method currently available. HISAT2 supports genomes of any size and has low memory requirements (approximately 4.3 GB of RAM for the human genome).

##### Assigning sequence reads to genomic features

For the purpose of assigning reads to genomic features (such as genes, exons, promoters and genomic bins) we used the software program FeatureCounts (v1.5.0-p1), from the Subread stand-alone package [18]. FeatureCounts is considerably faster than existing methods and has exceptionally low memory requirements, while being one of the top-ranking software in accuracy.

##### DE testing

The DE testing was performed with the R package EdgeR [19] using a modified Rscript from Su [20]. The Rscript was run through a Docker container that employs R version 3.3.0 and has been built with Bioconductor version 3.4 and all the required packages installed. The normalisation (for library size, gene length and sequencing depth) was performed on the raw count matrix produced by FeatureCounts using the Trimmed Mean of M-values (TMM) [21], which is set as the default normalisation method. A negative binomial generalized linear model (GLM) was fitted to the data and the testing procedure for determining differential expression was performed using quasi-likelihood (QL) F-test. The *P*-value adjustment was performed using the Benjamini-Hochberg method and in order to restrict the false discovery rate (FDR) to 0.05, all the genes with adjusted *P*-values less than 0.05 were selected. The filtering of the gene list (threshold default values: adjusted P-value 0.05 and |log2FC| ≥ 1) was performed with a custom python script.

##### Functional analysis

The functional pathway analysis was performed with the BioInfoMiner platform [22]. BioInfoMiner exploits biological hierarchical vocabularies through statistical and network analysis to detect and rank significantly altered processes and the hub linker genes involved. For our analysis we utilised Gene Ontology (GO) [23] and MGI Mammalian Phenotype Ontology [24]. BioInfoMiner maps the genes on a genomic network created from semantic data and prioritises them based on topological properties after minimising the impact of semantic noise (bias) through different types of correction. This analysis accomplishes the systemic interpretation of the complex cellular mechanisms described in the input gene lists, while at the same time it prioritises genes with central functional and regulatory roles in important cellular processes, underlying the studied phenotype. The correction for potential semantic inconsistencies of the selected vocabulary is implemented by linking the annotation of each gene with the ancestors of every direct correlated ontological term, consequently restoring the sound structure of an ontological tree. The BioInfoMiner platform is available online at the website https://bioinfominer.com.

### Genetic mapping

#### Genotyping

All CC lines were genotyped with high-density SNP markers using the MDA (620 K SNP markers), MUGA (7.7 K markers) and MegaMUGA (77 K markers) genotyping arrays based on the Illumina infinium platform. All SNPs with heterozygous or missing genotypes in the 8 CC founders or not common between the arrays were filtered out, leaving 170,935 SNPs. The SNPs were mapped onto build 37 of the mouse genome. A descent probability distribution was computed using the HAPPY HMM for each of the 170,935 SNPs intervals. The genotype status of the CC lines using the MUGA SNP was presented in CTC 2012 report [9, 25].

#### QTL mapping

The QTL mapping was based on the haplotype mosaic reconstruction with HAPPY. We developed two automated containerised R scripts using the mapping methodology proposed by Durrant and colleagues [26]. The first script uses the probability distribution of descent as calculated by the HAPPY algorithm to test for association between the reconstructed haplotype for each CC line at each locus and the median body weight gain at different time points. The second script estimates confidence intervals (CI) for each QTL through simulation of a QTL with a similar logP and strain effects in the neighbourhood of the observed QTL peak. We used the SNPtools R package to extract the genes inside the 95% CI for each QTL [27].

## Results

### Data stratification

It is well documented that obesity and obesity-related health complications are affected by gender, and that sex-specific differences have a genetic basis and cannot be solely attributed to differential hormonal regulation [28]. Gender-specific differences in adiposity as well as fat distribution, in addition to the distinctive genetic basis and hormonal regulation of men and women, may result in sex-specific patterns.

**Fig. 1:**
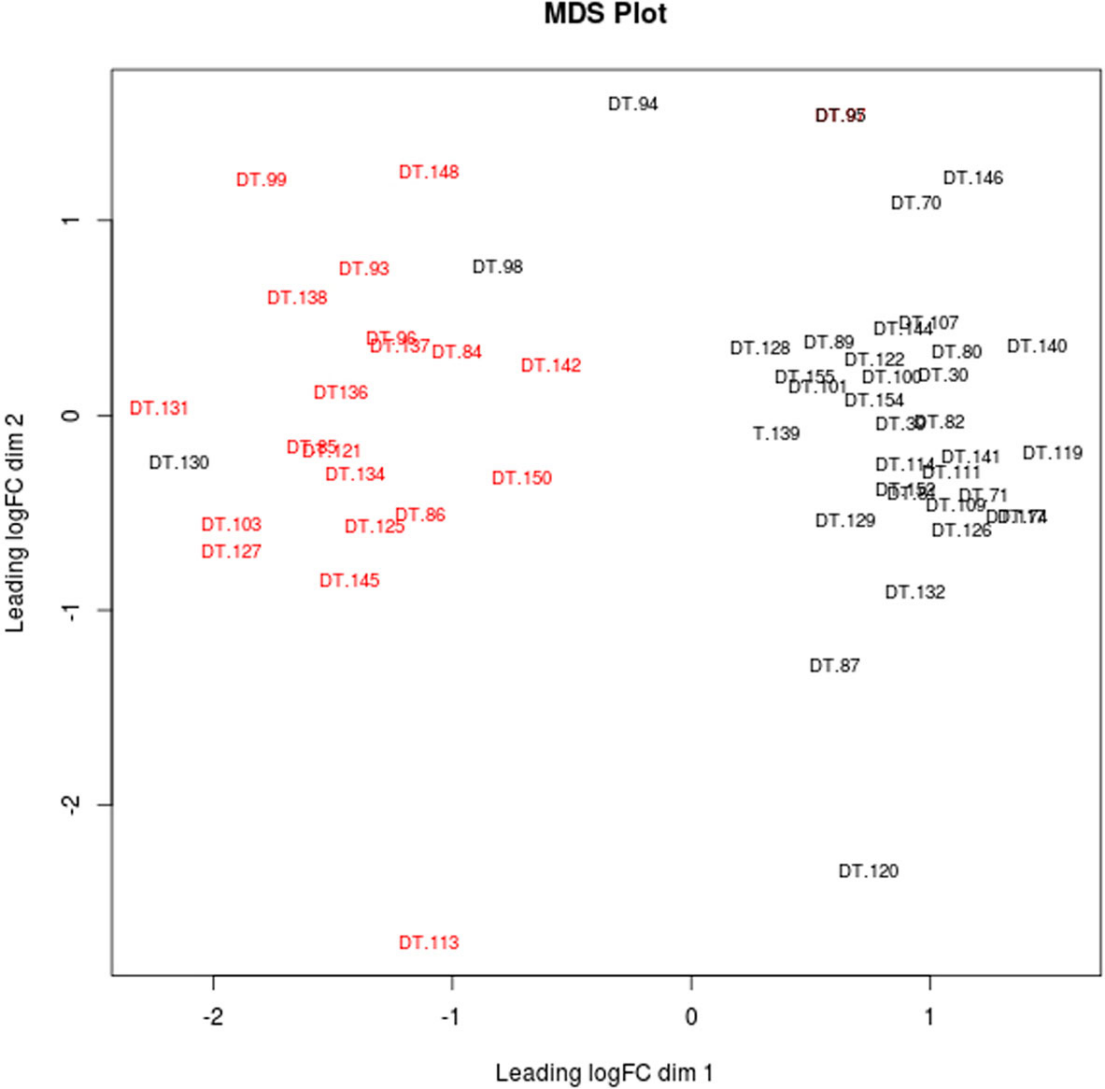
Multidimensional scaling (MDS) plot visualising the level of similarity of individual cases in the dataset. Male samples are represented with red, while female samples are represented with black labels

In order to assess the presence of confounding factors, we performed a multi-dimensional scaling analysis, using the EdgeR R package. According to the MDS plot (Fig. 1) the points cluster into two groups, not on the basis of the differences between the two conditions (“Normal” vs “Obese”) but on the basis of sex. This finding led to the conclusion that the observed separation in the principal component analysis performed, was the result of gender-specific differences, meaning that a straightforward approach would be prone to confounding biases.

In order to partition the samples into non-overlapping groups, we performed a stratified sampling dividing the total sample population into four strata, considering the sex and diabetes status of the mice. Of the total 85 samples, 21 samples were categorised in the Male-Nondiabetic group, 21 samples were categorised in the Male-Diabetic group, 36 samples were categorised in the Female-Nondiabetic group and 5 samples in the Female-Diabetic group. One sample (DT 123) was not used in the analysis due to missing data.

### QTL analysis

We performed haplotype association analysis, using the median value of the weight gain in the 12 weeks of the HFD challenge. We mapped one QTL on chr3 for female mice and one QTL on chr5 for male mice, designated as ObFL and ObML for obesity female locus and obesity male locus, respectively. Details on the position and size of each QTL are given in Table 1. We performed functional analysis with BioInfoMiner on the genes extracted from each of the QTLs using the Gene Ontology and MGI Mammalian Phenotype Ontology vocabularies.

**Table 1:** Positions of QTLs associated with obesity

QTL	Trait	Chr	logP	50% CI		90% CI		95% CI		Number of genes in 95% CI
				Position	Width (Mb)	Position	Width (Mb)	Position	Width (Mb)	
*ObFL*	ΔBW0–12	3	4.50	131.91–132.64	(0.72)	130.56–133.58	(3.02)	129.67–134.08	(4.41)	58
*ObML*	ΔBW0–12	5	4.37	51.08–55.46	(4.38)	45.78–61.14	(15.35)	44.01–62.37	(18.36)	120

*Chr* chromosome, *logP* negative 10-base logarithm of *P* value, *∆BW0–12* body weight change from week 0 to week 12. Positions and widths of the simulation-based 50, 90, and 95% CIs are given

#### Female mice body weight gain

In female mice, prioritised genes included *Sgms2* and *Hadh* that were found to be involved in various processes, such as increased energy expenditure (MP:0004889), decreased susceptibility to diet-induced obesity (MP:0005659) and increased circulating free fatty acid level (MP:0001554). Interestingly, *Sgms2* deficiency in mice increases insulin sensitivity and ameliorates high-fat diet-induced obesity and *Hadh*−/− mice, while having a disrupted β-oxidation pathway, are also protected from diet-induced obesity [29, 30]. Other prioritised genes are *Lef1*, *Dkk2* and *Egf*, which are all involved in the Wnt signaling pathway (GO:0016055). Non-canonical Wnt signaling has been shown to contribute to obesity-associated metabolic dysfunction by increasing adipose tissue inflammation [31].

#### Male mice body weight gain

In male mice the most highly prioritised gene is *Ppargc1a*, which is found to be involved in various enriched processes both in GO and in MGI Mammalian Phenotype Ontology, including decreased muscle weight (MP:0004232), regulation of muscle tissue development (GO:1901863) and lipid modification (GO:0030258). *Ppargc1a* is a transcriptional co-activator that regulates genes involved in energy metabolism through its interaction with Pparγ. In human GWAS analysis a single nucleotide variant of *Ppargc1a* (rs8192678) has been associated with susceptibility to obesity and insulin resistance [32]. Another important gene prioritised through both vocabularies, is *Cckar*, found involved in processes such as abnormal small intestinal transit time (MP:0006002) and abnormal intestinal cholesterol absorption (MP:0002645). Rats with a naturally occurring mutation in *Cckar* (Otsuka Long-Evans Tokushima Fatty (OLETF) rat) develop diabetes and obesity [33]. Prioritised gene *Sod3* is involved along with *Ppargc1a* in: response to reactive oxygen species (GO:0000302), increased susceptibility to injury (MP:0005165), and abnormal cytokine secretion (MP:0003009). Over-expression of *Sod3* in high-fat diet fed mice has been shown to block diet induced obesity [34]. *Med28* is involved in the regulation of muscle cell differentiation (GO:0051147). *Med28* is one of the subunits of the Mediator complex, which acts as a transcription factor co-activator and plays an important role in muscle metabolism by enhancing the transcriptional activity of *Ppargc1a* and *Pparα* [35]. Lastly, *Slit2* is also prioritised and shares enriched terms with *Ppargc1a*, such as regulation of smooth muscle cell migration (GO:0014910) and response to organonitrogen compound (GO:0010243). *Slit2* has been shown to regulate metabolic function and thermogenic activity and improve glucose homeostasis in diet-induced obese mice, mainly through *Ucp1* [36], which has been found highly under-expressed in male obese mice, in comparison to female obese mice in our analysis.

### RNA-seq analysis

DE analysis was performed on the Male-Nondiabetic and Female-Nondiabetic groups separately. In order to perform the statistical differential expression tests we divided the samples into two subgroups, 10 samples were categorised in the Male-Nondiabetic-Obese group (the male case group), 11 samples were categorised in the Male-Nondiabetic-Normal (the male control group) 12 samples were categorised in the Female-Nondiabetic-Obese group (the female case group) and 24 samples in the Female-Nondiabetic-Normal group (the female control group). Differences in library size were addressed by TMM normalisation.

#### Female nondiabetic normal vs female nondiabetic obese

As a first step we tried to assess the DE genes within the two genders, performing the DE tests between control and case groups of the same gender. The Female-Nondiabetic-Normal samples (control group) versus the Female-Nondiabetic-Obese samples (case group) DE genes list consisted of 1382 DE genes. Using BioInfoMiner for the enrichment analysis we obtained two prioritised gene list of 23 DE genes from GO and 21 DE genes from MGI Mammalian Phenotype. Seven genes were common in both gene lists. Processes that were highly enriched in DE genes in female mice include digestion (GO:0007586), regulation of insulin secretion (GO:0050796), response to lipid (GO:0033993), fatty acid biosynthetic process (GO:0006633), increased energy expenditure (MP:0004889), decreased susceptibility to diet-induced obesity (MP:0005659), abnormal glucose tolerance (MP:0005291) and decreased circulating leptin level (MP:0005668). Top prioritised, linker genes in female mice include *Lepr* and *Ppargc1a* that were under-expressed in obese females and *Pnlip*, *Pyy*, and *Ins2* that were over-expressed in obese females. Lepr functions as a receptor for leptin, an adipose secreted hormone that regulates energy expenditure, satiety, lipid and glucose metabolism and immune system activation. Pancreatic lipase is normally secreted from the pancreas and is efficient in the digestion of dietary fats. Inhibition of Pnlip may prevent HFD induced obesity in mice. Orlistat, an inhibitor of Pnlip was the first FDA-approved anti-obesity therapeutic drug in treating diet-induced obesity [37]. Increased pancreatic expression of *Pnlip* has been observed in mice induced by fasting via the PPARα-FGF21 signalling pathway [38]. PYY is synthesised and released from specialised cells found predominantly within the distal gastrointestinal tract and regulates appetite. Transgenic mice with increased circulating PYY are resistant to diet-induced obesity [39]. *Ins2* ectopic expression in the liver has been observed before in mice subjected to HFD [40]. We hypothesise that *Pyy* may be expressed ectopically in the liver in the same way as *Ins2* in response to the HFD.

#### Male nondiabetic normal vs male nondiabetic obese

The DE genes list for the Male-Nondiabetic-Normal samples (control group) versus the Male-Nondiabetic-Obese samples (case group) consisted of 1589 DE genes. We performed functional analysis on this list and obtained two prioritised gene lists of 24 DE genes from GO and 22 genes from MGI Mammalian Phenotype. Ten of the genes from the two prioritised gene lists were common in both. Enriched pathways in male mice are overwhelmingly related to muscle and cardiac muscle processes: impaired muscle contractility (MP:0000738), abnormal skeletal muscle mass (MP:0004817), myopathy (MP:0000751), cardiac muscle hypertrophy (GO:0003300). A second category of enriched processes involves ion transport (GO:0006811). Top prioritised linker genes in male mice include *Nos1*, *Ryr1*, *Des*, *Ttn* all under-expressed in obese males while *Ryr2* is over-expressed. *Ryr1* is mainly expressed in skeletal muscle. The encoded protein functions as a calcium release channel in the sarcoplasmic reticulum. However, there is a number of studies suggesting that *RyRs* are widely expressed and have been linked with inositol 1,4,5-trisphosphate receptors in hepatocytes [41]. Downregulation of *Ryr1* could lead to reduced release of Ca^2+^ from the sarcoplasmic (muscle cells) and the endoplasmic reticulum (hepatic cells) into the cytoplasm and therefore hinder muscle contraction as well as the regulation of mitochondrial metabolism and glycogen degradation [42, 43]. *Des* encodes a muscle-specific class III intermediate filament, which is important to help maintain the structure of sarcomeres and has been linked with hepatic fibrosis due to obesity [44]. *Titin* is an essential component of skeletal and cardiac muscles, but it has been suggested in recent studies that titin isoforms are expressed in non-muscle tissues including the liver, with an essential role in maintaining cellular organisation and contributing to signal transduction [45]. *Ryr2* is primarily expressed in cardiac muscle. Ryr2 channels are associated with mitochondrial metabolism, gene expression regulation and cell survival, in addition to their role in cardiomyocyte contraction. A recent study links *Ryr2* to insulin release and glucose homeostasis, suggesting that the upregulation of *Ryr2* might be a coping mechanism in response to stress [46]. Nos1 produces nitric oxide (NO), which has multiple biological functions. Downregulation of *Nos1* in obesity and diabetes is largely attributed to insulin resistance [47].

#### Male vs female comparisons

Finally, in order to assess the DE genes between the two genders we performed DE tests between groups that belonged to different genders. The DE genes list for the Male-Nondiabetic-Normal samples versus the Female-Nondiabetic-Normal samples comparison, consisted of 1923 genes. Functional analysis produced two prioritised gene lists consisting of 28 genes from GO and 25 genes from MGI Mammalian Phenotype, with 9 common genes. As for the DE genes for the Male-Nondiabetic-Obese samples versus the Female-Nondiabetic-Obese samples, the genes list consisted of 1940 DE genes. Functional analysis produced two prioritised gene lists consisting of 40 genes from GO and 22 genes from MGI, with 8 common genes. Between the two groups 482 DE genes are shared while 1441 genes are uniquely DE in the Male-Nondiabetic-Normal versus the Female-Nondiabetic-Normal samples and 1458 genes are uniquely DE in the Male-Nondiabetic-Obese versus the Female-Nondiabetic-Obese samples.

The 482 DE genes that are common in the two comparisons are enriched in genes that are involved in processes such as the epoxygenase P450 pathway (GO:0019373), long-chain fatty acid metabolic process (GO:0001676), negative regulation of gluconeogenesis (GO:0045721), increased circulating gonadotropin level (MP:0003362), and regulation of NF-κB import into nucleus (GO:0042345). Functional analysis of these 482 DE expressed genes produced 21 prioritised genes from GO and 20 prioritised genes from MGI Mammalian Phenotype. Of these 8 genes were common in both lists. *Cav1*, *Egfr, Gstp1, Gcg*, and *Nox4* are consistently over-expressed in male versus female mice both in the non-obese and obese comparisons. Caveolin-1 regulates hepatic lipid accumulation and glucose metabolism and plays an important role in metabolic adaptation [48]. Hepatic glucagon action has been associated with elevated fatty acid oxidation and might act protectively against non-alcoholic fatty liver disease (NAFLD) [49]. *Esr1*, *Il1b* and *Ptgs2* are consistently over-expressed in female versus male mice in both comparisons. *Ptgs2* is involved in inflammation in fat and drives obesity-linked insulin resistance and fatty liver [50]. *Tph1* is under-expressed in non-obese males and over-expressed in obese males in comparison to females. In contrast, *Ryr2* is over-expressed in non-obese males and under-expressed in obese males in comparison to females. Tph1 is involved in the synthesis of serotonin, which is known to modulate appetite, energy expenditure and thermogenesis. Tph1-deficient mice fed a high-fat diet are protected from obesity, insulin resistance and NAFLD [51].

Functional analysis of the 1441 genes that were uniquely DE in the Male-Nondiabetic-Normal versus the Female-Nondiabetic-Normal comparison produced enriched processes like abnormal bone volume (MP:0010874), increased mesenteric fat pad weight (MP:0009298), unsaturated fatty acid and lipid metabolic process (GO:0033559, GO:0006629), regulation of hormone levels (GO:0010817), and a number of terms related to inflammatory processes, such as abnormal IgG2a level (MP:0020176), increased susceptibility to autoimmune diabetes (MP:0004803) and small intestinal inflammation (MP:0003306). The list of prioritised genes consisted of 21 genes from GO and 23 genes from MGI, with 9 overlapping genes. Genes involved in inflammatory processes, such as *Il2, Il4, Il10*, and *Il1r1*, were predominantly over-expressed in female mice. *Vdr* and *Lepr* were also over-expressed in females. Males had over-expressed *Mrap2* and *Oprm1*. *Mrap2* encodes a protein that modulates melanocortin receptor signalling. Mice deficient in Mrap2 exhibit severe obesity and a mutation in this gene may be associated with severe obesity in human patients [52].

**Fig. 2:**
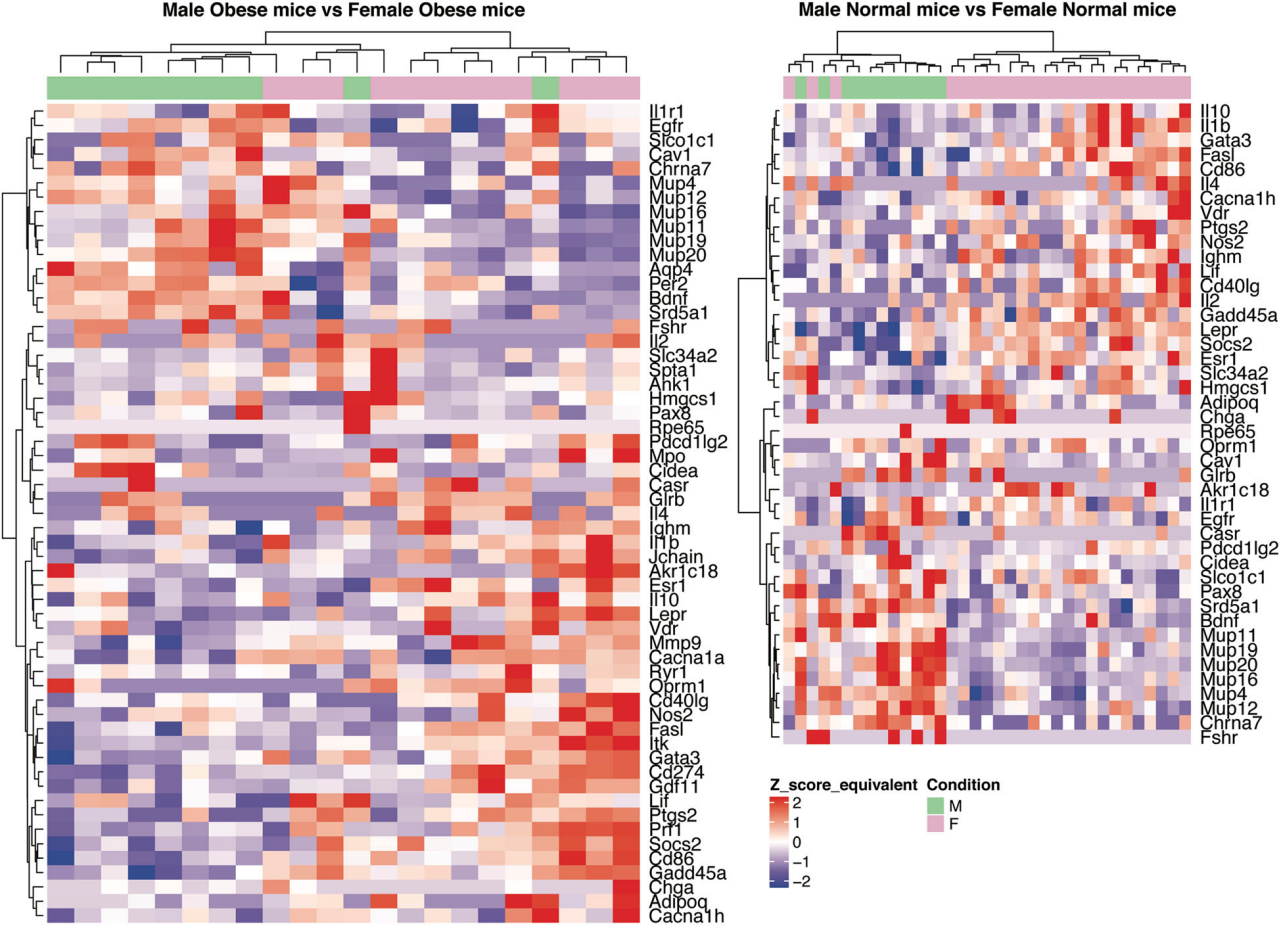
Heatmap graphical representation of Male Obese mice versus Female Obese (right). The genes (y axis) are derived from the union of the prioritised lists produced

Finally, functional analysis of the 1458 unique DE genes from the Male-Nondiabetic-Obese versus the Female-Nondiabetic-Obese comparison resulted again in enriched immune response related processes (GO:0006955) and positive regulation of inflammatory response (GO:0050729) with related genes also predominantly over-expressed in obese females. The list of prioritised genes consisted of 34 genes from GO and 20 genes from MGI Mammalian Phenotype, with 4 overlapping genes. Genes related to immunological processes include *Ifng*, *Il6*, and *Tnf*. Other enriched processes were muscle system processes (GO:0003012) and abnormal muscle contractility (MP:0005620), abnormal adrenaline level (MP:0003962), abnormal blood pH regulation (MP:0003027), digestion (GO:0007586) and decreased glucagon secretion (MP:0002711). Genes involved in these processes were predominantly under-expressed in male mice, for example, the *Mc2r* and *Pyy*. Notably, both *Ins1* and *Ins2* are also highly over-expressed in obese female mice compared to an almost zero level of expression in male mice. Mc2r belongs to the MCR family that plays an important role in appetite and energy regulation via leptin signalling. Increased MCRs expression in the liver has been associated with muscle tissue damage and potentially exerts a protective effect through metabolic regulation [53]. The common prioritised genes identified by both GO and MGI Mammalian Phenotype ontologies have been used to produce two heatmaps of male versus female comparisons which are presented in Fig. 2.

## Discussion

Obesity encompasses a complex array of traits closely related to the development of type 2 diabetes, metabolic syndrome and associated with comorbidities such as cardiovascular disease, hypertension, atherosclerosis and various cancers. In this study we have used a systems biology approach to examine genetic susceptibility and hepatic gene response to obesity in CC mice. The CC panel is a unique resource with wide genetic diversity that enables complex trait genomic mapping at a very high resolution. Moreover, it provides the opportunity to study differences in genetic expression in a diverse genetic reference population under controlled environmental conditions.

To facilitate this analysis, we developed a unified, automated workflow using Docker containers that can be simultaneously deployed on different computation infrastructures and can facilitate cross-platform collaboration. This is achieved by creating an identical insulated and stable operating environment that can be precisely controlled resulting in consistency overtime, without the worry of conflicting dependencies, UNIX compatibility issues and system updates. Docker containers can be used effectively to address some of the major setbacks of microarray and next generation sequencing technologies. The fast start-up time of Docker containers opens up the ability to chain together the execution of multiple containers and the development of sophisticated workflows for genomic analyses.

This work takes into consideration the overall analytical performance of the workflow as well as computational cost. Our aim was to develop an automated, accurate, and computationally efficient analytical process, while keeping the computational requirements at a minimum. Exploiting the minimal performance loss introduced by the Docker engine we developed a modular architecture for the integrative analysis of QTL and RNA-seq data that can be run on a personal computer (with approximately 4 GB of RAM or higher). For the aforementioned purpose the bioinformatic tools have been picked according to their overall effectiveness and their computational cost as described in the methods. The workflow we propose requires no more than 4.3 GB of RAM, 10 GB of physical drive and can run a top-down analysis of 20 GB of input data in less than 3 h (these values may change depending on whether multiprocessing or multi-threading are used).

Using our workflow, we mapped two QTLs related to the body weight gain phenotype, one on chromosome 5 for male mice and one on chromosome 3 for female mice. The QTL on chromosome 5 has already been described as significant in relation to the percentage of body fat, in response to an atherogenic diet, where *Ppargc1a* was suggested to be a candidate gene through comparative genomics and haplotype analysis [54]. There has also been evidence that suggests Ppargc1a activity may be significantly influenced by sex, although results to that direction have been contradictory with the rs8192678 allele influencing the risk of developing obesity in men but not in women, while in female mice *Ppargc1a* expression is estrogen regulated and has a protective effect against obesity-induced oxidative damage [55, 56]. In our analysis *Ppargc1a* is under-expressed in obese versus normal females. Additional genes involved in pathways closely related to the obesity phenotype highlighted by our analysis as potential candidates that play a role in male mice susceptibility to obesity are *Cckar*, *Sod3*, *Med28*, and *Slit2*.

QTLs on chromosome 3 have been previously described to be involved in the percentage of body fat and heat loss, without the introduction of a high fat diet [57, 58]. To our knowledge, this is the first time that the two mapped QTL are described in a sex specific context. It is notable that while the QTL and DE do not overlap significantly, they converge at the functional level, pointing to potentially common regulatory mechanisms. For example, in female mice there is a single differentially expressed gene located inside the QTL on chr3, *Cyp2u1*, a hydroxylase that is involved in the metabolism of long chain fatty acids. However, the two gene lists derived from the QTL and differential expression analysis share a high number of common relevant enriched pathways, such as increased energy expenditure (MP:0004889), decreased susceptibility to diet-induced obesity (MP:0005659) and increased circulating free fatty acid level (MP:0001554).

The comparison of male and female DE results (presented in supplementary Tables 3 and 4) showed that male and female mice have completely different mechanisms of response to HFD induced obesity. Sex differences in glucose metabolism have been previously described in mouse strains. Males have been found to be more prone to developing insulin resistance and obesity than females after being fed a high-fat / high carbohydrate diet. In addition, the same study showed that estrogen contributes to insulin sensitivity in females, and testosterone exacerbates insulin resistance in C57BL/6 J mice [59].

In our results, ectopic expression of *Pyy* and *Ins2* in the liver appear to be coping mechanisms utilised by female obese mice but not males. We also observed extrapancreatic expression of *Pnlip* in female obese mice. A hepatic to pancreatic switch that occurs in a compensatory mode under stress conditions has been previously described [60]. Under-expression of *Lepr* indicates liver resistance to leptin, which leads to impaired hepatic insulin sensitivity, regulation of lipid metabolism and glucose homeostasis in female obese mice [61, 62]. Our findings regarding the cases of male nondiabetic obese mice when compared with male nondiabetic normal mice indicate that obesity in the male population heavily affects genes related to the skeletal and cardiac tissues, which agrees with previous studies connecting obesity with impaired muscular structure and function, as well as abnormal regulation of insulin secretion and glucose homeostasis [46, 63, 64]. Diet induced obesity has been shown to alter skeletal muscle fiber types in male but not in female mice [65].

**Fig. 3:**
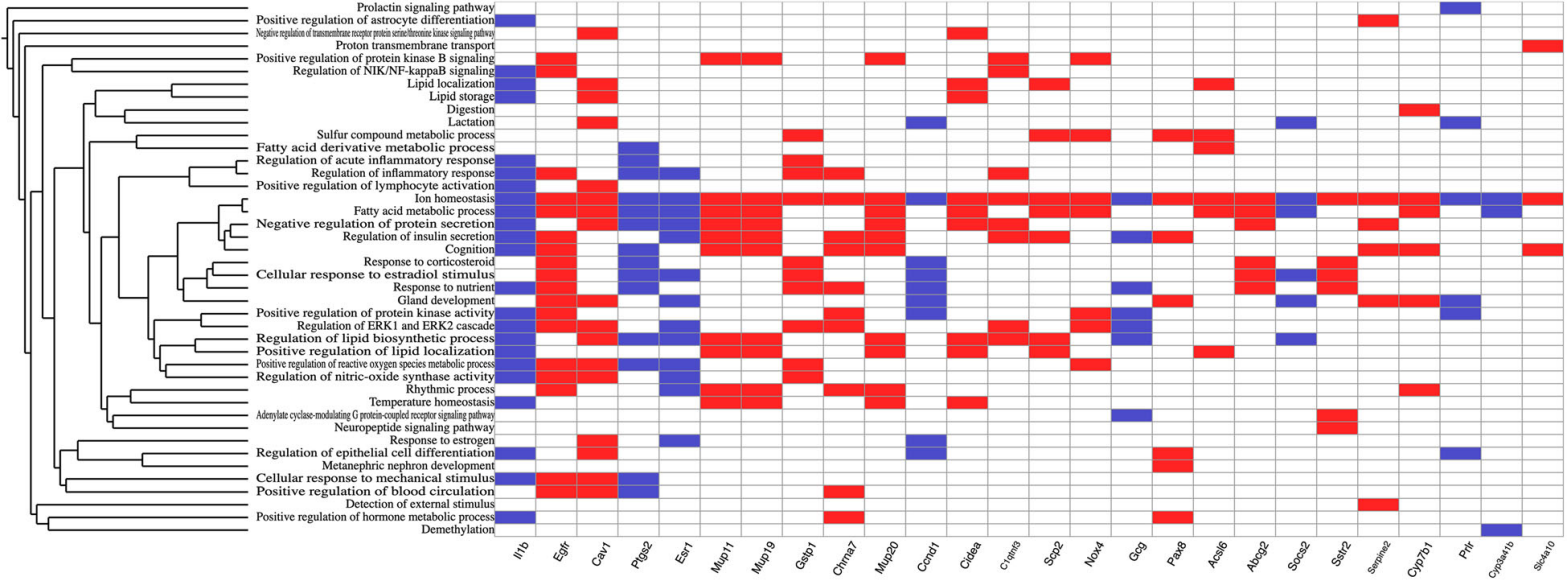
Heatmap graphical representation of the most highly prioritised genes that are common in Male vs Female comparisons and their respective ontologies based on GO produced by BioInfoMiner. Genes marked blue are overexpressed in Female mice while genes marked red are overexpressed in Male mice

A direct comparison of DE genes in female versus male mice subjected to HFD (the complete lists of DE genes of Male-Nondiabetic-Normal vs Female-Nondiabetic-Normal and Male-Nondiabetic-Obese vs Female-Nondiabetic-Obese, are available in Supplementary Tables 5 and 6, respectively) confirms gender specific differences in the responses of both resistant and susceptible individuals. Male mice appear to undergo and respond to oxidative stress induced by the HFD by over-expressing antioxidant enzymes such as *Gstp1* and *Nox4* (Fig. 3). They also respond to the metabolic stress they undergo by over-expressing metabolism modulating genes, such as *Cav1* (Fig. 3). Additionally, as illustrated in Fig. 3, female mice over-express genes involved in the regulation of the inflammatory response, such as *Il1b*, in comparison to male mice. A previous study of the sex-dependent inflammatory response of HFD fed mice has shown that male mice tend to develop low-grade systemic inflammation, while female mice are protected through expansion of their population of anti-inflammatory T lymphocytes [66]. As systemic low-grade inflammation caused by obesity is believed to play a role in insulin resistance, it is of great interest to further look into sex-dependent inflammatory responses and coping mechanisms.

Our work addresses data integration from analytical platforms at the genomic and trancsriptomic level, but we expect that our findings can be extended to accommodate broader integration schemes and result in a variety of computational genomic analysis pipelines. Moreover, our findings highlight the potential of Docker technologies for the development of prototype analytical workflows that suit the individual needs of different research objectives. As a future step in this project, we plan to develop a fully automated, dockerised workflow to perform eQTL analysis on Collaborative Cross mice, which is a significantly more computationally demanding task and will greatly benefit from the possibility of fast and reliable remote deployment.

## Conclusions

We observed that the genetic mechanisms which underlie susceptibility and response to HFD induced obesity differ in female and male mice. This clear distinction in the systemic response to the HFD challenge and obesity between male and female mice points to the need for further research into distinct sex-related mechanisms in metabolic disease in humans as well.

Moreover, the integration of data using ontological functional analysis, which showed that different genes but similar pathways are involved in the genetic susceptibility and are disturbed in diet induced obesity, demonstrate the importance of integrating genomic and transcriptomic data at the level of signaling pathways rather than focusing on genes. This way the collective background biological knowledge is meaningfully utilised to systemically interpret results at the genomic scale.

## Supplementary Information

**Additional file 1:** **Table S1.** Median values of baseline body weight of mice at the start of the dietary challenge and body weight gain after 12 weeks of dietary challenge for each CC-line.

**Additional file 2:** **Table S2.** CC line, sex, baseline body weight (BW0) at the start of the dietary challenge, body weight gain after 12 weeks (ΔBW0-12) of dietary challenge, aNA blood glucose levels at T180 after IPGTT challeng (BGM-T180) for all mice included in the analysis.

**Additional file 3:** **Table S3.** List of differentially expressed genes from the female non-diabetic normal vs. female non-diabetic obese mice with the logFC and adjusted *p*-values.

**Additional file 4:** **Table S4.** List of differentially expressed genes from the male non-diabetic normal vs. male non-diabetic obese mice with the logFC and adjusted *p*-values.

**Additional file 5:** **Table S5.** List of differentially expressed genes from the male non-diabetic normal vs. female non-diabetic normal mice with the logFC and adjusted *p*-values.

**Additional file 6:** **Table S6.** List of differentially expressed genes from the male non-diabetic obese vs. female non-diabetic obese mice with the logFC and adjusted *p*-values.

## Data Availability

The dataset(s) supporting the conclusions of this article is (are) available in the GEO (and SRA) repositories, under the accession number GSE126490 at https://www.ncbi.nlm.nih.gov/geo/query/acc.cgi?acc=GSE126490. All docker images used in this analysis are freely available on Docker Hub at enios/rnaseq-qtl repository, https://hub.docker.com/r/enios/rnaseq-qtl/. Accompanying Python scripts and documentation are available on Github at e-nios/RNA-seq-QTL repository, https://github.com/e-nios/RNA-seq-QTL. The genotype data of the CC mouse lines are available on the UNC Systems Genetics website, http://csbio.unc.edu/CCstatus/index.py.

## Abbreviations

CC: Collaborative Cross
GRP: Genetic reference panel
HFD: High-fat diet
RIL: Recombinant inbred lines
BMI: Body mass index
QTL: Quantitative trait loci
CPU: Central processing unit
TAU: Tel-Aviv University
SE: Standard error
IPGTT: Intraperitoneal glucose tolerance test
IP: Intraperitoneal
RIN: RNA Integrity Number
log2FC: log2 fold change
GO: Gene ontology
MGI: Mouse genome informatics
CI: Confidence interval
MDS: Multidimensional scaling
DE: Differential expression / differentially expressed
GWAS: Genome-wide association study
Sgms2: Sphingomyelin synthase 2
Hadh: Hydroxyacyl-coenzyme A dehydrogenase
Lef1: Lymphoid enhancer binding factor 1
Dkk2: Dickkopf WNT signaling pathway inhibitor 2
Egf: Epidermal growth factor
Ppargc1a: Peroxisome proliferative activated receptor, gamma, coactivator 1 alpha
Cckar: Cholecystokinin A receptor
Sod3: Superoxide dismutase 3
Med28: Mediator complex subunit 28
Slit2: Slit homolog 2
Ucp1: Uncoupling protein 1
Lepr: Leptin receptor
Pnlip: Pancreatic lipase
Pyy: Peptide YY
Ins1: Insulin I
Ins2: Insulin II
Nos1: Nitric oxide synthase 1
Ryr1: Ryanodine receptor 1
Ryr2: Ryanodine receptor 2
Des: Desmin
Ttn: Titin
Cav1: Caveolin 1
Egfr: Epidermal growth factor receptor
Gstp: Glutathione S-transferase
Gcg: Glucagon
Nox4: NADPH oxidase 4
NAFLD: Non-alcoholic fatty liver disease
Esr1: Estrogen receptor 1
Il1b: Interleukin 1 beta
Ptgs2: Prostaglandin-endoperoxide synthase 2
Tph1: Tryptophan hydroxylase 1
Il2: Interleukin 2
Il4: Interleukin 4
Il6: Interleukin 6
Il10: Interleukin 10
Il1r: Interleukin 1 receptor
Vdr: Vitamin D receptor
Mrap2: Melanocortin 2 receptor accessory protein 2
Oprm1: Opioid receptor, mu 1
Ifng: Interferon gamma
Tnf: Tumour necrosis factor
Mc2r: Melanocortin 2 receptor
MCR: Melanocortin receptor
Cyp2u1: Cytochrome P450 family 2 subfamily U member 1
